# AZITHROMYCIN IN ACNE: A PROTAGONIST FOR FIXED DRUG REACTION?

**DOI:** 10.4103/0019-5154.41661

**Published:** 2008

**Authors:** Pratik Gahalaut, Emy Alexander

**Affiliations:** *From Department of Dermatology, Sri Ram Murti Smarak Institute of Medical Sciences, Bareilly, Uttar Pradesh, India. E-mail: drpratikg@hotmail.com*; 1 *From Department of Dermatology, Christian Medical College, Ludhiana, Punjab, India*

Azithromycin is a semi-synthetic macrolide derivative approved for treating mild to moderate infections of the skin, soft tissues, lower and upper respiratory tracts.^1^ Pulse azithromycin therapy^2^,^3^ is being increasingly used nowadays as a safe and effective treatment of acne vulgaris with excellent patient compliance. A hitherto unreported probable fixed drug eruption to azithromycin is being reported here.

A 26-year-old married female presented to the Dermatology outpatient Department with history and clinical features suggestive of acne vulgaris of 3-year duration. The patient denied taking any medication, either systemic or topical, for acne or any other disease in the preceding 2 months. She was started on azithromycin 500 mg, thrice weekly along with topical benzoyl peroxide application. Fifteen days afterwards, the patient came for follow-up with the complaint of a hyperpigmented lesion on the upper lip of 1-week duration. According to the patient, the lesion appeared spontaneously 6 days after starting azithromycin therapy and it worsened, i.e. became darker in colour on taking the next weekly azithromycin dose. However, she continued with the treatment and took the second pulse also. Although the lesion was asymptomatic at onset, she complained of itching in the lesion after taking a second pulse of azithromycin. She denied taking any other oral medication concurrently. On clinical examination, a well-circumscribed hyperpigmented macule, of size 1 × 1 cm^2^, was seen at the mucocutaneous junction of the upper lip on the left side. A provisional diagnosis of a probable fixed drug reaction (FDE) to azithromycin was made. The offending drug was stopped and the lesion improved with a short course of topical mild corticosteroid application. The obviously disturbed patient refused to undergo an oral provocation test again or to get the lesion biopsied.

Fixed drug eruptions, first described by Brocq in 1894, is one of the commonest types of adverse cutaneous drug reactions.^4^ They are responsible for 10% of all adverse drug reactions and occur in all ages, more so in young adults.^5^ FDE consists of recurrent eruptions characterized by sharply marginated, round, erythematous to violaceous macules that subsequently evolve into a plaque. Vesicles and haemorrhagic bullae with crusting may develop later on. Generalized bullous fixed drug eruption is rare, but severe. The lesions vary in size and can occur on any part of the skin and mucous membranes.^5^ Classically, the transitional epithelium of mucocutaneous junctions is involved, as in this case. In a study done by Gupta *et al.*, the lips were the most commonly involved site followed by genitals, arms, abdomen, hands and face.^6^ The diagnostic hallmark is its recurrence at previously affected sites with repeated ingestion of the suspected drug.^5^ Oral rechallenge test is still the only reliable method to confirm the causative agent, but unlike our patient, patient's co-operation is essential.^6^ When the acute phase subsides, there is usually residual hyperpigmentation that becomes more pronounced after each recrudescence. Although most commonly, non-steroidal anti-inflammatory drugs (NSAIDs) and antibiotics, namely sulphonamides, are implicated in FDE, over a 100 drugs are known to induce FDE.^5^ With the advent of new drugs, the list of offending drug is growing everyday.

An extensive search of the literature on Medline and internet failed to report FDE to azithromycin. Although FDE to azithromycin could not be confirmed in this case, probability of the same cannot be ruled out. Inducing awareness among physicians about this hitherto unreported side effect of azithromycin warrants intense observation and caution, since more such cases may be reported with extensive usage of this molecule in future.
